# A Single Nucleotide Polymorphism rs1010816 Predicts Sorafenib Therapeutic Outcomes in Advanced Hepatocellular Carcinoma

**DOI:** 10.3390/ijms24021681

**Published:** 2023-01-14

**Authors:** Chih-Lang Lin, Kung-Hao Liang, Ching-Chih Hu, Cheng-Hung Chien, Li-Wei Chen, Rong-Nan Chien, Yang-Hsiang Lin, Chau-Ting Yeh

**Affiliations:** 1 Liver Research Unit, Keelung Chang Gung Memorial Hospital, Keelung 204, Taiwan; 2 Liver Research Center, Chang Gung Memorial Hospital, Taoyuan 333, Taiwan; 3 Community Medicine Research Center, Keelung Chang Gung Memorial Hospital, Keelung 204, Taiwan; 4 College of Medicine, Chang Gung University, Taoyuan 333, Taiwan; 5 Department of Medical Research, Taipei Veterans General Hospital, Taipei 112, Taiwan; 6 Institute of Food Safety and Health Risk Assessment, National Yang-Ming Chiao-Tung University, Taipei 112, Taiwan; 7 Institute of Biomedical Informatics, National Yang-Ming Chiao-Tung University, Taipei 112, Taiwan

**Keywords:** genome-wide association study (GWAS), advanced hepatocellular carcinoma (aHCC), overall survival (OS)

## Abstract

Sorafenib is currently a targeted agent widely used in the treatment of advanced hepatocellular carcinoma (aHCC). However, to date there is still a lack of a reliable marker capable of predicting sorafenib therapeutic responses. Here, we conducted a genome-wide association study (GWAS) to identify candidate single-nucleotide polymorphism outcome predictors in aHCC patients. A total of 74 real-world sorafenib-treated aHCC patients were enrolled for GWAS and outcome analysis. GWAS showed that rs1010816 (*p* = 2.2 × 10^−7^) was associated with sorafenib therapeutic response in aHCC patients. Kaplan–Meier analysis indicated that the “TT” genotype was significantly associated with a favorable therapeutic response but not significantly associated with overall survival (OS). Univariate followed by multivariate Cox proportional hazard analysis showed that ascites, main portal vein thrombosis, lower platelet count, lower total sorafenib doses, higher PALBI score in model A and higher ALBI grade in model B were significantly associated with a shorter OS. Subgroup analysis showed that only in alcoholic aHCC patients treated by sorafenib, rs1010816 “TT” genotype was significantly associated with longer OS (*p* = 0.021). Sorafenib had a favorable therapeutic outcome in alcoholic aHCC patients carrying rs1010816 “TT” genotype.

## 1. Introduction

Globally, hepatocellular carcinoma (HCC) accounts for more than 80% of the histological subtypes of primary liver cancer, and it occurs more frequently in male gender, with one in 45 men compared with one in 113 women. HCC ranks the sixth in cancer incidence and the fourth in cancer-related death in the world [1,2]. Multiple etiological factors for HCC are involved, including chronic hepatitis B and C virus infections, alcohol addiction, metabolic liver diseases, and exposure to dietary toxins such as aflatoxin and aristolochic acid [3,4,5,6]. If HCC diagnosis is made in an early stage, curative surgical resection or non-surgical local ablation therapy (such as radiofrequency ablation) can be performed to achieve complete remission. Liver transplantation is considered the best therapeutic modality in selected patients who fulfill designated criteria, resulting in total removal of the tumor and adjacent microinvasion, and the cirrhotic liver entirely replaced [7,8]. In contrast, in patients with incurable HCC, such as those in advanced stages or with distant metastases, the recommended treatments are largely palliative, including transcatheter arterial chemoembolization for intermediate stage (Barcelona clinic liver cancer [BCLC] stage B) patients, and radiotherapy, chemotherapy, targeted therapy, and immunotherapy for patients in a further advanced stage (BCLC stage C) [9,10,11]. Recently, atezolizumab was identified as an immune checkpoint inhibitor through interaction of programmed death-ligand 1. Bevacizumab was a monoclonal antibody to block vascular endothelial growth factor (VEGF) activity [12]. Atezolizumab plus bevacizumab treatment has been suggested as a first-line therapy in HCC [13]. A comparative clinical study demonstrated that the combined atezolizumab plus bevacizumab treatment was correlated with better survival outcomes of advanced unresectable HCC patients than those with only sorafenib treatment [14]. To date, if immunotherapy is not available, tyrosine kinase inhibitors such as sorafenib and lenvatinib are still recommended as the first-line treatments for advanced HCC (aHCC), and regorafenib and cabozantinib are recommended as the second-line therapies [15,16,17].

Among these targeted agents in the current treatment guidelines, sorafenib was the first approved and is the most widely used targeted drug for patients with advanced-stage HCC. According to the results of two large-scale randomized controlled studies, SHARP (Sorafenib HCC assessment randomized protocol) and Asia Pacific (AP), sorafenib can prolong the overall survival (OS) in patients with incurable HCC, albeit the therapeutic outcomes and long-term prognosis vary widely among patients and the outcomes remain unsatisfactory [18,19]. In fact, sorafenib failed to show statistically significant benefits in aHCC patients with extrahepatic metastasis or macroscopic vascular invasion in a recent subgroup and exploratory analysis [20]. Additionally, unlike occasional complete remission (0–44%) in HCC patients receiving systemic chemotherapy or hepatic arterial infusion chemotherapy, sorafenib rarely resulted in complete remission in a recent study in which only 12 of 1119 (1%) patients were classified as complete responders [11,21,22,23,24,25]. Therefore, to overcome these problems, it is necessary to determine a reliable marker capable of predicting sorafenib therapeutic responses so that clinicians can carefully select favorable patients to receive sorafenib therapy and, to advise unfavorable patients to choose other novel effective treatments.

In this study, in order to achieve this purpose, we have conducted a pilot genome-wide association study (GWAS) to identify candidate single-nucleotide polymorphism (SNP) markers as a prognosis predictor for sorafenib therapeutic response in BCLC stage C HCC patients.

## 2. Results

### 2.1. Basic Demographic Characteristics

A total of 74 aHCC patients were included. Their basic clinical characteristics are listed in Table 1. The ECOG performance status in most patients was in stage 0. Etiology analysis showed that 44 patients were HBsAg positive (59.5%), 23 were anti-HCV positive (31.1%), and 24 were alcoholic (32.4%). Notably, it is not uncommon for patients to have more than one etiological factor. There were 62 men (83.8%) and 12 women (16.2%) and the mean age of male patients and female patients were 57.0 ± 10.5 years and 60.7 ± 10.0 years, respectively. The mean age of 74 aHCC patients was 57.6 ± 10.5 years. The largest tumor size was 6.1 ± 4.1 cm, 66 patients (89.2%) had histological or clinical evidence of liver cirrhosis, 17 (23%) had ascites, and 56 (75.7%) had a history of previous treatment. In addition, higher percentages of initial metastasis (42, 56.8%) and portal vein thrombosis (45, 60.8%) were also found, suggesting a far advanced stage of HCC.

### 2.2. Genetic Associations with Respect to Objective Responses to Sorafenib Therapy

**Figure 1:**
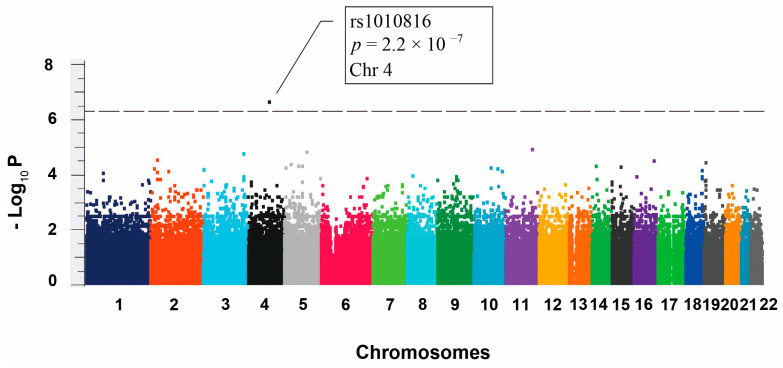
Manhattan plot for sorafenib therapeutic response in advanced HCC patients. The association values in –log_10_ *p* values are shown by chromosome. The chromosomes are shown by different color and labeled with number from 1 to 22. A genome-wide significant association signal is observed and rs1010816 is the top hit. The threshold *p* value (5 × 10^−7^) is displayed as dash line.

**Figure 2:**
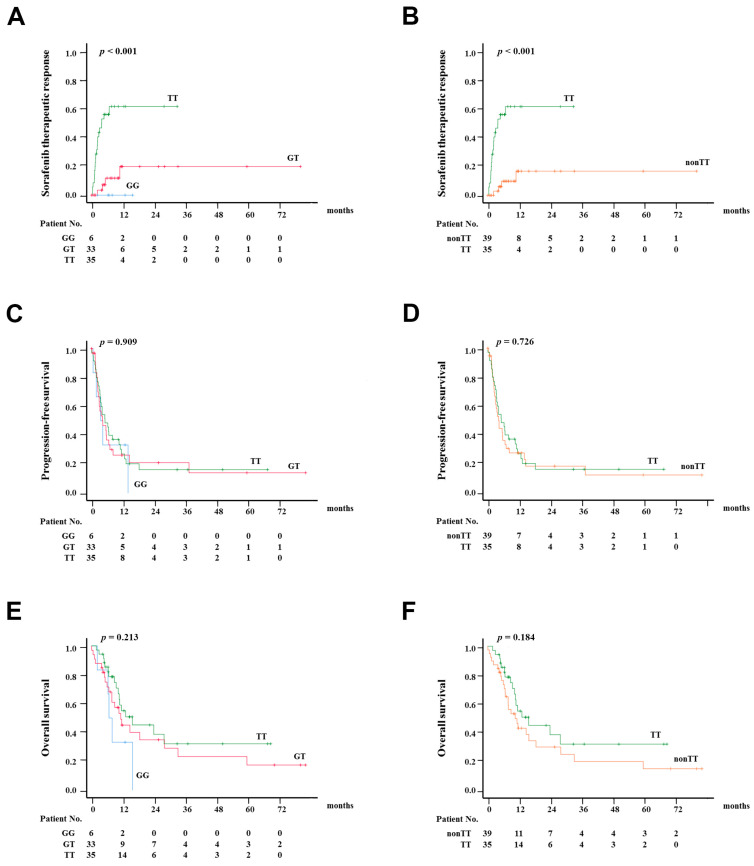
Kaplan–Meier analysis in advanced HCC patients receiving sorafenib therapy. (**A**) Sorafenib therapeutic response (time-to-response) in different rs1010816 genotypes. (**B**) Sorafenib therapeutic response (time-to-response) in rs1010816 “TT” and “non-TT” genotypes. (**C**) Progression-free survival in different rs1010816 genotypes. (**D**) Progression-free survival in rs1010816 “TT” and “non-TT” genotypes. (**E**) Overall survival in different rs1010816 genotypes. (**F**) Overall survival in rs1010816 “TT” and “non-TT” genotypes.

A genome-wide screen was conducted on this retrospective cohort of 74 subjects, who either had positive sorafenib therapeutic response (*n* = 24) or no response (*n* = 50). Figure 1 shows that the top hit rs1010816 (*p* = 2.2 × 10^−7^) was associated with genome-wide significance for sorafenib therapeutic response in aHCC patients. Accordingly, rs1010816 was selected for further investigation. Based on the Hardy–Weinberg equilibrium, there are 6, 33 and 35 persons with the GG, GT and TT genotypes at rs1010816, respectively. Accordingly, Kaplan–Meier analysis was used to examine the significance of the therapeutic response, progression-free survival (PFS) and OS for the rs1010816 genotype in aHCC patients receiving sorafenib treatment (Figure 2). It was found that the “TT” genotype was significantly associated with shorter time-to-therapeutic response (i.e., a better response) when compared with those in “GT” and “GG” genotypes (*p* < 0.001) (Figure 2a). Subsequently, because the number of patients with “GG” genotype was small, the rs1010816 genotype was reclassified as rs1010816 “TT” and “non-TT”. As expected, the “TT” genotype was significantly associated with shorter time-to-response when compared with that of the “non-TT” genotype (*p* < 0.001) (Figure 2b). However, there was no significant difference among the rs1010816 genotypes in PFS (Figure 2c; *p* = 0.909) and in OS (Figure 2e; *p* = 0.213). In addition, no significant difference was found between the “TT” and “non-TT” genotypes in PFS (Figure 2d; *p* = 0.726) and OS (Figure 2f; *p* = 0.184). On the other hand, Kaplan–Meier analysis showed that the Child–Pugh grade was significantly correlated with progression free survival and overall survival of aHCC patients, but not in sorafenib therapeutic response (Supplementary Figure S1).

### 2.3. Identification of Genetic and Clinical Predictors for Sorafenib Therapeutic Response

Univariate followed by multivariate Cox proportional hazard analysis was performed to analyze the association between genetic and clinical factors and the therapeutic response (calculated as time-to-response) after receiving sorafenib (Table 2). It was found that the rs1010816 genotype (GG vs. GT vs. TT) (*p* < 0.001), rs1010816 “TT” (*p* < 0.001), age (*p* = 0.033), and sorafenib doses use (*p* = 0.002) were associated with time-to-sorafenib therapeutic response. However, after being adjusted for confounding factors, multivariate analysis revealed that only the rs1010816 genotype (*p* = 0.003 in model A and *p* = 0.002 in model B) was an independent predictor.

### 2.4. Identification of Genetic and Clinical Predictors for Progression-Free Survival

Subsequently, univariate followed by multivariate Cox proportional hazard analysis was performed to understand the genetic and clinical factors associated with PFS (Table 3). It was found that the presence of ascites (*p* = 0.030), bilirubin (*p* = 0.002), total sorafenib doses (*p* < 0.001), Child–Pugh score (*p* = 0.003), Child–Pugh grade (*p* = 0.001), and PALBI grade (*p* = 0.011) were associated with PFS. However, after being adjusted for confounding factors, multivariate analysis revealed that the independent predictors were the presence of ascites (*p* = 0.042) and sorafenib total doses (*p* < 0.001) in model A and total sorafenib doses (*p* < 0.001) in model B.

### 2.5. Identification of Genetic and Clinical Predictors for Overall Survival

Finally, univariate followed by multivariate Cox proportional hazard analysis was performed to understand the genetic and clinical factors associated with OS (Table 4). It was found that the presence of ascites (*p* <0.001), main portal vein thrombosis (*p* = 0.043), albumin (*p* = 0.007), bilirubin (*p* = 0.003), platelet count (*p* = 0.043), total sorafenib doses (*p* < 0.001), Child–Pugh score (*p* < 0.001), Child–Pugh grade (*p* < 0.001), ALBI score (*p* = 0.004), ALBI grade (*p* = 0.015), PALBI score (*p* = 0.006) and PALBI grade (*p* < 0.001) were associated with OS. However, after being adjusted for confounding factors, multivariate analysis revealed that the independent predictors were the presence of ascites (*p* = 0.024), main portal vein thrombosis (*p* = 0.002), platelet count (*p* = 0.035), total sorafenib doses (*p* < 0.001), and PALBI score (*p* = 0.015) in model A. Furthermore, multivariate analysis revealed that the independent predictors were the presence of ascites (*p* = 0.008), main portal vein thrombosis (*p* = 0.004), platelet count (*p* = 0.011), total sorafenib doses (*p* < 0.001), and ALBI grade (*p* = 0.010) in model B.

### 2.6. Subgroup Analysis to Identify Patient Subgroups wherein rs1010816 Genotype Effectively Predicted Overall Survival

**Figure 3:**
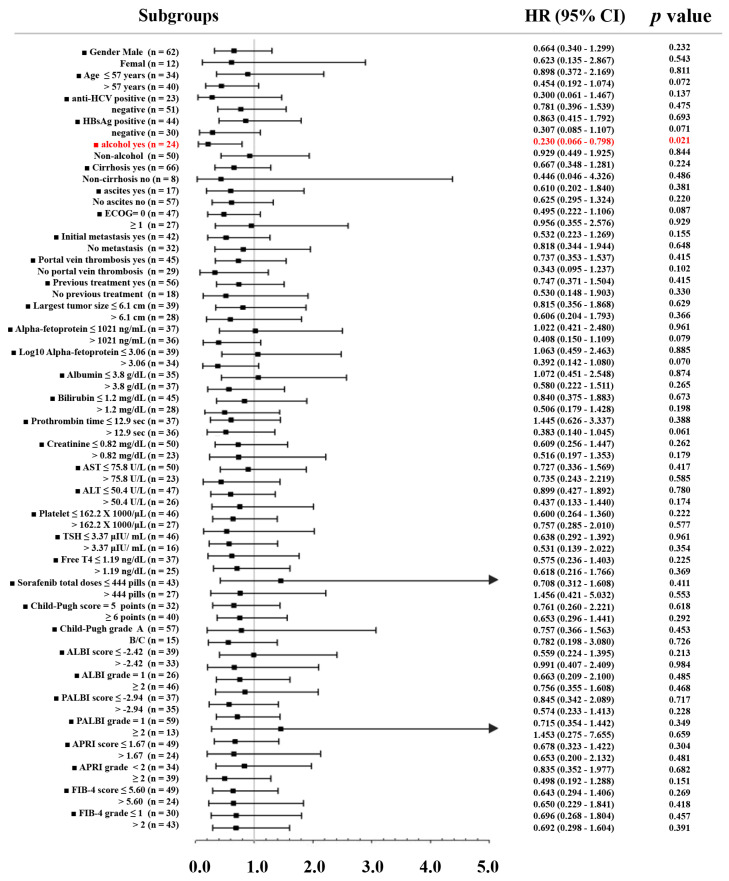
Forest plot analysis for overall survival in relation to the rs1010816 “TT” genotype in different clinical subgroups of sorafenib-treated patients. The mean values of clinical parameters were used as cutoffs. Red values indicate statistical significance *p* < 0.05. HR—hazardous ratio; CI—confidence interval.

To gain more insight into why the rs1010816 genotype was associated with therapeutic response but not OS, we divided patients into different clinical subgroups for further analysis (Figure 3). It was discovered that only in aHCC patients with alcoholism, was the “TT” genotype significantly associated with longer OS (*p* = 0.021), implying that alcohol-related aHCC might affect the predictive role of rs1010816 in OS.

## 3. Discussion

In the present study, our goal was to identify pretherapeutic biomarkers capable of predicting the outcomes of sorafenib treatment, so that only individuals with a higher probability of responses and favorable outcomes should be treated with this drug. Through GWAS, it was found that there was a linkage disequilibrium in the SNPs surrounding the rs1010816 associated with sorafenib treatment response. There were 6, 33 and 35 persons with the GG, GT and TT genotypes at rs1010816, respectively. Furthermore, multivariate analysis also showed that the rs1010816 genotype was the only independent predictor for time-to-sorafenib therapeutic response. However, the rs1010816 “TT” genotype was not significantly associated with favorable PFS or OS, as compared with those with rs1010816 “non-TT” genotype. Multivariate analysis revealed that the presence of ascites or sorafenib total dose was significantly associated with PFS. So far, information on the rs1010816 genotype is limited. The SNP rs1010816 was located on chromosome 4 and between the LOC105377364 and RNU6-289P genes. Accordingly, we proposed that the SNP rs1010816 was considered as intergenic SNP. Based on the single-nucleotide polymorphism database at the NCBI, the data showed that the frequency of the rs1010816 TT genotype in a global population (n = 147080) was approximately 74%. The presence of ascites, main portal vein thrombosis, platelet count, total sorafenib doses, PALBI score, or ALBI grade were independent predictors for OS. We were puzzled by this unexpected result. Therefore, a subgroup analysis was subsequently performed, and it was found that the “TT” genotype was significantly associated with longer OS in patients with alcoholism. In other words, the rs1010816 “TT” genotype in alcoholic aHCC patients receiving sorafenib therapies was associated with a favorable sorafenib therapeutic outcome (in both therapeutic response and OS). Previous studies showed that SNPs could modulate several cellular functions through alteration of mRNA secondary structure, modulation of RNA alternative splicing and regulation of target-gene-translation efficiency [26]. We proposed that SNP rs1010816 may regulate the expression of LOC105377364 via potential underlying mechanisms mentioned above, leading to regulated hepatic alcohol metabolism. The functional roles of SNP rs1010816/LOC105377364/sorafenib response axis in HCC need to be determined in the future.

To date, although immunotherapy has been recommended as the first-line treatment for advanced HCC patients with vascular invasion and/or extrahepatic metastasis for some time, sorafenib is still the most widely used standard-of-care first-line treatment for these patients [7,8]. Sorafenib significantly improved OS in aHCC patients but the outcomes varied greatly among different individuals. Several clinical prognostic factors have been proposed. The recently published GIDEON (global investigation of therapeutic decisions in hepatocellular carcinoma and of its treatment with sorafenib) was a large-scale global prospective observational registration study that recruited 3202 patients with unresectable HCC treated in real-life practice conditions to evaluate the use and tolerability of sorafenib [27]. It was reported that the type and incidence of drug-related adverse events and those leading to sorafenib discontinuation were similar in patients with Child–Pugh A and B liver function. However, survival was longer in patients with Child–Pugh A liver function (13.6 months) than Child–Pugh B (5.2 months) and Child–Pugh C patients (2.6 months) in the intent-to-treat analysis. Subsequently, among the individual components of the Child–Pugh score, albumin levels, ascites, and bilirubin levels all have important prognostic values for OS. In addition, McNamara et al. [28] have reported a meta-analysis study that included four randomized controlled trials and twenty-six cohort studies with a total of 8678 patients. Multivariable meta-regression analysis revealed that patients with ECOG performance status of 2 (*p* = 0.04) and Child–Pugh B (*p* = 0.001) were significantly negatively associated with OS.

However, in our real-world cohort study, although the Child–Pugh score/grade remained significant in univariate analysis (*p* < 0.001, Table 4), the Child–Pugh stage/grade and ECOG performance status were not significant predictors of OS. Furthermore, a multivariate analysis showed that the presence of ascites, main portal vein thrombosis, lower platelet count, lower total sorafenib doses, and higher PALBI score in model A and higher ALBI grade in model B were significantly associated with a shorter OS, while the Child–Pugh score was not included as an independent predictor. Therefore, the Child–Pugh score was not considered a good predictor for OS in our patients, but the ALBI grade or PALBI grade may appear to be a better marker. Johnson et al. [29] proposed the ALBI grade in 2015, which used a different liver-dysfunction-assessment model to better select HCC patients receiving sorafenib and only involved the serum albumin and bilirubin levels from 1313 patients in Japan initially, and subsequently validated the results in 5097 patients in six cohorts from other geographic regions. In addition, the ALBI grade could eliminate the need for subjective variables such as ascites and encephalopathy, which is the requirement of the standard Child–Pugh grade. Regarding the comparison with the sub-categories according to points [5,6,7,8,9,10,11,12,13,14,15] in the Child-Pugh classification in the original publication, the combination of Child-Pugh A as the inclusion criteria and ALBI as the stratification factor in trials of systemic therapy may allow HCC patients to have more extraordinary discrimination ability when treated with sorafenib [30]. The PALBI grade was proposed by Roayaie based on the ALBI grade in 2015 [31]. By contrast, although the calculation of ALBI or PALBI grade was considered complicated, they avoided the disadvantages of the “ceiling effect” or “floor effect” through arbitrary cut-off values. Moreover, both ALBI and PALBI could ensure accurate assessment of liver function and predict the prognosis in HCC patients [32,33].

Our study has some limitations. First, the predictive value of rs1010816 genotypes in sorafenib therapeutic response or in atezolizumab plus bevacizumab treatment should be evaluated using independent cohorts of patients in the future. Second, this study was a retrospective investigation in a single center. These clinical findings should be verified by prospective studies. Recently, deep learning has become a powerful tool for patient stratification in precision medicine [34]. If we have more clinical information about rs1010816 genotypes on sorafenib therapeutic response, effective deep learning methods can be applied for evaluating the prognosis of aHCC patients based on rs1010816 genotypes.

In this study, we discovered a single-nucleotide polymorphism rs1010816, which could effectively predict the therapeutic response of sorafenib in patients with aHCC. However, it could not predict OS. It was possible that its inability to predict OS was largely due to the heterogeneity of cancer etiologies and patient populations. A reasonable approach to solve this problem was to determine the pre-treatment biomarker in various patient subgroups. Hence, it was found that only in patients with alcoholic aHCC, was the “TT” genotype significantly associated with longer OS during sorafenib treatment.

## 4. Materials and Methods

### 4.1. Patients

This retrospective cohort study was conducted under the approval of the Institutional Review Board of Chang Gung Medical Hospital. From February 2008 to January 2018, a total of 74 consecutive aHCC patients with BCLC stage C were enrolled, who were not suitable for curative therapy and received sorafenib therapy in Linkou Chang Gung Medical Hospital, and their samples were retrievable from a tissue bank. The diagnosis criteria of HCC included dynamic computed tomography or dynamic magnetic resonance imaging plus angiography when the HCC size in cirrhotic liver exceeded 2 cm with typical HCC characteristics. When such typical features were not present, a liver biopsy or aspiration cytology diagnosis was required for diagnosis.

The clinical parameters were retrospectively retrieved from medical records, including gender, age, HBV surface antigen (HBsAg), antibody against HCV (anti-HCV), alcoholism, liver cirrhosis status, presence of ascites, Child–Pugh classification, Eastern Cooperative Oncology Group (ECOG) performance status, initial metastasis, main portal vein thrombosis, previous treatments, largest tumor size (in diameter), total sorafenib doses, and date of last follow-up or HCC related death. Biochemistry and hemogram analysis included alpha-fetoprotein, albumin, bilirubin, aspartate transaminase (AST), alanine transaminase (ALT), creatinine, thyroid-stimulating hormone (TSH), free thyroxine (free T4), prothrombin time, and platelet count. In addition, the APRI (aspartate aminotransferase-to- platelet ratio index) [35], ALBI (Albumin-Bilirubin) grade [29], FIB-4 (Fibrosis-4) score [36], and PALBI (Platelet-Albumin-Bilirubin) grade were calculated according to their formulas.

In order to evaluate the therapeutic response of sorafenib, alpha-fetoprotein (AFP) changes and modified response evaluation criteria in solid tumors (mRECIST) were applied as previously described [37,38,39]. Accordingly, a 20% reduction of AFP was also considered as achieving treatment response [38]. OS was calculated from the date of sorafenib treatment to the date of death or the last follow-up.

### 4.2. Genome-Wide Variant Assessment

The Affymetrix Axiom Genome-Wide TWB 2.0 array plates, designed jointly by the Taiwan Biobank, the National Center for Genome Medicine (Nankang, Taipei, Taiwan) and the Thermo Fisher Scientific (Waltham, MA, USA), were used for assessing ~750,000 genome-wide variants in this GWAS study. The base-calling algorithm was Affymetrix APT. The hybridization of samples onto the microarrays and the washing procedure were conducted following the manufacturer’s protocol. The Affymetrix scanner scanned the fluorescent signals and then stored them as digital images for further data processing. Associations with the phenotype were evaluated using Fisher’s exact test of allele counts.

### 4.3. Statistical Analysis

Parametric data were recorded as mean ± standard deviation and compared using a two-sample t-test. Dichotomized data were recorded as numbers and percentages (%) and compared using the Chi-square test or Fisher’s exact tests, where appropriate. Univariate followed by multivariate Cox proportional hazard models were performed to estimate the therapeutic response and progression-free survival (PFS) and OS for clinical factors and genotypic variables. In this study, significant factors determined from the univariate analysis were included for multivariate Cox proportional hazards. The Kaplan–Meier method was performed to estimate the survival probability between the different groups, and the log-rank test was performed to compare survival. All tests were two-tailed, and a *p* < 0.05 was considered statistically significant. All statistical analyses were performed using Statistical Package for the Social Sciences (SPSS) statistics Version 20 (SPSS, Chicago, IL, USA).

## 5. Conclusions

In summary, we demonstrated that the rs1010816 “TT” genotype could be an effective predictor of favorable therapeutic response and OS in alcoholic aHCC treated by sorafenib. Prospective studies should be conducted to verify this result.

## Figures and Tables

**Table 1:** Clinical characteristics of patients included.

Clinical Parameter	All Patients (*n* = 74)
Gender, male, *n* (%)	62 (83.8%)
Age (All patients), years, mean ± SD	57.6 ± 10.5
rs1010816 (GG/GT/TT), *n* (%)	6/33/35 (8.1%/44.6%/47.3%)
Anti-HCV, positive, *n* (%)	23 (31.1%)
HBsAg, positive, *n* (%)	44 (59.5%)
Alcoholism, yes, *n* (%)	24 (32.4%)
Cirrhosis, yes, *n* (%)	66 (89.2%)
Ascites, yes, *n* (%)	17 (23%)
ECOG, 0/1/2/3, *n* (%)	47/24/2/1 (63.5%/32.4%/2.7%/1.4%)
Initial metastasis, yes, *n* (%)	42 (56.8%)
Portal vein thrombosis, yes, *n* (%)	45 (60.8%)
Previous treatment, yes, *n* (%)	56 (75.7%)
Largest tumor size, cm, mean ± SD	6.1 ± 4.1
Alpha-fetoprotein, ng/mL, median (range)	1021 (2 to 831,318)
Log_10_ Alpha-fetoprotein, mean ± SD	3.06 ± 1.85
Albumin, g/dL, mean ± SD	3.8 ± 0.5
Bilirubin, mg/dL, mean ± SD	1.2 ± 1.3
Prothrombin time, sec, mean ± SD	12.9 ± 1.1
Creatinine, mg/dL, mean ± SD	0.82 ± 0.37
AST, U/L, mean ± SD	75.8 ± 56.1
ALT, U/L, mean ± SD	50.4 ± 38.9
Platelet, 1000/μL, mean ± SD	162.2 ± 100.8
TSH, μIU/mL, mean ± SD	3.37 ± 3.47
Free T4, ng/dL, mean ± SD	1.19 ± 0.5
Sorafenib total doses, pills, mean ± SD	444 ± 403
Child-Pugh score, mean ± SD	5.9 ± 1.2
Child-Pugh grade, A/B/C, *n* (%)	57/14/1 (79.2%/19.4%/1.4%)
ALBI score, mean ± SD	−2.42 ± 0.54
ALBI grade, 1/2/3, *n* (%)	26/45/1 (36.1%/62.5%/1.4%)
PALBI score, mean ± SD	−2.94 ± 0.53
PALBI grade, 1/2/3, *n* (%)	59/9/4 (81.9%/12.5%/5.6%)
APRI score, mean ± SD	1.67 ± 1.57
APRI grade, 0/1/2/3, *n* (%)	23/11/20/19 (31.5%/15.1%/27.4%/26%)
FIB-4 score, mean ± SD	5.60 ± 4.93
FIB-4 grade, 0/1/2, *n* (%)	7/23/43 (9.6%/31.5%/58.9%)

Abbreviations: ECOG—Eastern Cooperative Oncology Group; AST—aspartate aminotransferase; ALT—alanine aminotransferase; TSH—thyroid-stimulating hormone; free T4—free thyroxine; ALBI—Albumin-Bilirubin; PALBI—Platelet-Albumin-Bilirubin; APRI—aspartate aminotransferase-to- platelet ratio index; FIB-4—Fibrosis-4.

**Table 2:** Cox proportional hazard analysis for association between genetic and clinical factors and the sorafenib therapeutic response.

Clinical Parameter	Univariate Analysis		Multivariate Analysis ^@model A^		Multivariate Analysis ^#model B^	
	Hazard Ratio (95% CI)	*p*	Hazard Ratio (95% CI)	*p*	Hazard Ratio (95% CI)	*p*
rs1010816 (GG/0 vs. GT/1 vs. TT/2)	7.093 (2.487–20.231)	**<0.001**	5.288 (1.794–15.583)	**0.003**		
rs1010816 (non-TT/0 vs. TT/1)	7.980 (2.709–23.505)	**<0.001**			5.955 (1.912–18.549)	**0.002**
Gender, male = 1	0.653 (0.317–1.348)	0.249				
Age, per year increase	0.961 (0.926–0.997)	**0.033**	0.961 (0.923–1.001)	0.053	0.961 (0.923–1.001)	0.057
Anti-HCV, positive = 1	0.520 (0.194–1.394)	0.194				
HBsAg, positive = 1	1.563 (0.668–3.656)	0.303				
Alcoholism, yes = 1	1.726 (0.763–3.904)	0.190				
Cirrhosis, yes = 1	1.092 (0.256–4.649)	0.905				
Ascites, yes = 1	0.682 (0.203–2.295)	0.537				
ECOG, per increase	0.480 (0.191–1.205)	0.118				
Initial metastasis, yes = 1	1.138 (0.504–2.569)	0.755				
Portal vein thrombosis, yes = 1	1.424 (0.619–3.276)	0.405				
Previous treatment, yes = 1	0.905 (0.359–2.283)	0.832				
Largest tumor size, cm	1.020 (0.911–1.141)	0.732				
Log_10_ Alpha-fetoprotein per increase	0.972 (0.735–1.285)	0.840				
Albumin, per g/dL increase	1.151 (0.527–2.513)	0.725				
Bilirubin, per mg/dL increase	0.690 (0.332–1.431)	0.319				
Prothrombin time, per sec increase	1.141 (0.791–1.645)	0.480				
Creatinine, per mg/dL increase	0.741 (0.181–3.037)	0.677				
AST, per U/L increase	0.999 (0.990–1.009)	0.843				
ALT, per U/L increase	1.004 (0.996–1.012)	0.368				
Platelet, per 1000/μL increase	0.999 (0.994–1.003)	0.577				
TSH, perμIU/mL increase	0.936 (0.790–1.109)	0.443				
Free per T4, ng/dL increase	1.033 (0.482–2.212)	0.933				
Sorafenib total doses, per pills increase	1.001 (1.000–1.002)	**0.002**	1.001 (1.000–1.002)	0.054	1.001 (1.000–1.002)	0.058
Child-Pugh score, per increase	0.724 (0.424–1.236)	0.236				
Child-Pugh grade, per increase	0.252 (0.036–1.792)	0.169				
ALBI score, per increase	0.817 (0.368–1.812)	0.619				
ALBI grade, per increase	0.647 (0.288–1.450)	0.290				
PALBI score, per increase	0.679 (0.281–1.645)	0.391				
PALBI grade, per increase	0.270 (0.040–1.833)	0.180				
APRI score, per increase	0.969 (0.742–1.265)	0.817				
APRI score, per increase	0.903 (0.640–1.276)	0.564				
FIB-4 score, per increase	0.952 (0.861–1.053)	0.341				
FIB-4 grade, per increase	0.952 (0.517–1.753)	0.874				

Abbreviations: ECOG—Eastern Cooperative Oncology Group; AST—aspartate aminotransferase; ALT—alanine aminotransferase; TSH—thyroid-stimulating hormone; free T4—free thyroxine; ALBI—Albumin-Bilirubin; PALBI—Platelet-Albumin-Bilirubin; APRI—aspartate aminotransferase-to- platelet ratio index; FIB-4—Fibrosis-4. Bold values indicate statistical significance *p* < 0.05; @model A: rs1010816 (GG vs. GT vs. TT), age, and sorafenib total doses were included for multivariate Cox proportional hazard analysis; #model B: rs1010816TTnonTT (non TT vs. TT), age, and sorafenib total doses were included for multivariate Cox proportional hazard analysis.

**Table 3:** Cox proportional hazard analysis for association between genetic and clinical factors and progression free survival.

Clinical Parameter	Univariate Analysis		Multivariate Analysis ^@model A^		Multivariate Analysis ^#model B^	
	Hazard Ratio (95% CI)	*p*	Hazard Ratio (95% CI)	*p*	Hazard Ratio (95% CI)	*p*
rs1010816 (GG/0 vs. GT/1 vs. TT/2)	0.916 (0.611–1.374)	0.672				
rs1010816 (non-TT/0 vs. TT/1)	0.911 (0.542–1.533)	0.727				
Gender, male = 1	1.224 (0.854–1.755)	0.271				
Age, per year increase	1.009 (0.984–1.035)	0.492				
Anti-HCV, positive = 1	1.007 (0.569–1.782)	0.980				
HBsAg, positive = 1	0.933 (0551–1.580)	0.795				
Alcoholism, yes = 1	0.834 (0.476–1.461)	0.525				
Cirrhosis, yes = 1	0.578 (0.246–1.357)	0.208				
Ascites, yes = 1	2.035 (1.069–3.874)	**0.030**	2.235 (1.029–4.854)	**0.042**	1.959 (0.972–3.946)	0.060
ECOG, per increase	1.212 (0.840–1.747)	0.303				
Initial metastasis, yes = 1	0.771 (0.452–1.313)	0.338				
Portal vein thrombosis, yes = 1	0.805 (0.472–1.375)	0.428				
Previous treatment, yes = 1	1.916 (0.985–3.726)	0.055				
Largest tumor size, cm	0.986 (0.913–1.063)	0.707				
Log_10_Alpha-fetoprotein per increase	1.154 (0.965–1.379)	0.116				
Albumin, per g/dL increase	0.741 (0.441–1.244)	0.257				
Bilirubin, per mg/dL increase	1.441 (1.140–1.820)	**0.002**	1.399 (0.992–1.973)	0.055	1.362 (0.971–1.911)	0.074
Prothrombin time, per sec increase	0.983 (0.762–1.268)	0.896				
Creatinine, per mg/dL increase	0.997 (0.440–2.262)	0.995				
AST, per U/L increase	1.003 (0.996–1.009)	0.466				
ALT, per U/L increase	0.999 (0.992–1.006)	0.770				
Platelet, per 1000/μL increase	1.002 (0.999–1.005)	0.115				
TSH, perμIU/mL increase	0.975 (0.896–1.061)	0.555				
Free per T4, ng/dL increase	1.238 (0.735–2.086)	0.423				
Sorafenib total doses, per pills increase	0.998 (0.997–0.999)	**<0.001**	0.998 (0.997–0.999)	**<0.001**	0.998 (0.997–0.999)	**<0.001**
Child-Pugh score, per increase	1.523 (1.150–2.015)	**0.003**	0.837(0.547–1.282)	0.414		
Child-Pugh grade, per increase	2.994 (1.610–5.567)	**0.001**			0.891 (0.396–2.007)	0.781
ALBI score, per increase	1.462 (0.842–2.539)	0.177				
ALBI grade, per increase	1.447 (0.832–2.519)	0.191				
PALBI score, per increase	1.820 (0.937–3.538)	0.077				
PALBI grade, per increase	2.233 (1.198–4.163)	**0.011**	1.018 (0.368–2.821)	0.972	0.923 (0.340–2.506)	0.875
APRI score, per increase	0.957 (0.798–1.147)	0.634				
APRI score, per increase	0.926 (0.743–1.154)	0.493				
FIB-4 score, per increase	0.998 (0.942–1.057)	0.940				
FIB-4 grade, per increase	0.722 (0.486–1.074)	0.108				

Abbreviations: ECOG—Eastern Cooperative Oncology Group; AST—aspartate aminotransferase; ALT—alanine aminotransferase; TSH—thyroid-stimulating hormone; free T4—free thyroxine; ALBI—Albumin-Bilirubin; PALBI—Platelet-Albumin-Bilirubin; APRI—aspartate aminotransferase-to- platelet ratio index; FIB-4—Fibrosis-4. Bold values indicate statistical significance *p* < 0.05; @model A: ascites, bilirubin, sorafenib total doses, Child-Pugh score, and PALBI grade were included for multivariate Cox proportional hazard analysis; #model B: ascites, bilirubin, sorafenib total doses, Child-Pugh grade, and PALBI grade were included for multivariate Cox proportional hazard analysis.

**Table 4:** Cox proportional hazard analysis for association between genetic and clinical factors and overall survival.

Clinical Parameter	Univariate Analysis		Multivariate Analysis ^@model A^		Multivariate Analysis ^#model B^	
	Hazard Ratio (95% CI)	*p*	Hazard Ratio (95% CI)	*p*	Hazard Ratio (95% CI)	*p*
rs1010816 (GG vs. GT vs. TT)	0.674 (0.423–1.074)	0.097				
rs1010816 (non-TT vs. TT)	0.662 (0.359–1.221)	0.187				
Gender, male = 1	0.950 (0.632–1.429)	0.806				
Age, per year increase	1.010 (0.980–1.042)	0.508				
Anti-HCV, positive = 1	0.557 (0.267–1.165)	0.120				
HBsAg, positive = 1	1.646 (0.864–3.135)	0.130				
Alcoholism, yes = 1	0.984 (0.513–1.888)	0.961				
Cirrhosis, yes = 1	0.549 (0.214–1.410)	0.213				
Ascites, yes = 1	3.433 (1.791–6.579)	**<0.001**	2.445 (1.122–5.328)	**0.024**	2.958 (1.333–6.564)	**0.008**
ECOG, per increase	1.334 (0.882–2.019)	0.172				
Initial metastasis, yes = 1	0.879 (0.481–1.607)	0.675				
Portal vein thrombosis, yes = 1	1.942 (1.020–3.696)	**0.043**	3.526 (1.580–7.866)	**0.002**	3.127 (1.427–6.853)	**0.004**
Previous treatment, yes = 1	1.385 (0.674–2.846)	0.375				
Largest tumor size, cm	1.004 (0.930–1.083)	0.925				
Log_10_Alpha-fetoprotein per increase	1.192 (0.978–1.453)	0.082				
Albumin, per g/dL increase	0.420 (0.223–0.791)	**0.007**				
Bilirubin, per mg/dL increase	1.356 (1.109–1.660)	**0.003**				
Prothrombin time, per sec increase	0.890 (0.654–1.210)	0.457				
Creatinine, per mg/dL increase	1.958 (0.915–4.190)	0.083				
AST, per U/L increase	1.005 (0.998–1.012)	0.163				
ALT, per U/L increase	0.990 (0.978–1.002)	0.096				
Platelet, per 1000/μL increase	1.003 (1.000–1.006)	**0.043**	1.004 (1.000–1.007)	**0.035**	1.005 (1.001–1.008)	**0.011**
TSH, perμIU/mL increase	1.044 (0.965–1.129)	0.288				
Free per T4, ng/dL increase	1.074 (0.426–2.709)	0.879				
Sorafenib total doses, per pills increase	0.997 (0.995–0.998)	**<0.001**	0.997 (0.995–0.998)	**<0.001**	0.997 (0.995–0.998)	**<0.001**
Sorafenib treatment response ≤ 120 days, yes = 1	0.466 (0.216–1.008)	0.052				
Child-Pugh score, per increase	1.840 (1.419–2.388)	**<0.001**				
Child-Pugh grade, per increase	4.142 (2.103–5.567)	**<0.001**				
ALBI score, per increase	2.671 (1.379–5.173)	**0.004**				
ALBI grade, per increase	2.322 (1.179–4.575)	**0.015**			2.2570 (1.256–5.259)	**0.010**
PALBI score, per increase	2.919 (1.351–6.306)	**0.006**	2.287 (1.173–4.457)	**0.015**		
PALBI grade, per increase	3.466 (1.850–6.493)	**<0.001**				
APRI score, per increase	0.999 (0.803–1.242)	0.989				
APRI score, per increase	0.915 (0.700–1.197)	0.517				
FIB-4 score, per increase	1.044 (0.979–1.113)	0.185				
FIB-4 grade, per increase	0.774 (0.481–1.244)	0.290				

Abbreviations: ECOG—Eastern Cooperative Oncology Group; AST—aspartate aminotransferase; ALT—alanine aminotransferase; TSH—thyroid-stimulating hormone; free T4—free thyroxine; ALBI—Albumin-Bilirubin; PALBI—Platelet-Albumin-Bilirubin; APRI—aspartate aminotransferase-to-platelet ratio index; FIB-4—Fibrosis-4. Bold values indicate statistical significance *p* < 0.05; @model A: ascites, portal vein thrombosis, albumin, bilirubin, platelet, sorafenib total doses, Child–Pugh score, ALBI score, and PALBI score were included for multivariate Cox proportional hazard analysis. #model B: ascites, Portal vein thrombosis, albumin, bilirubin, platelet, sorafenib total doses, Child–Pugh grade, ALBI grade, and PALBI grade were included for multivariate Cox proportional hazard analysis.

## Data Availability

The data presented in this study are available upon request from the corresponding author.

## Supplementary Materials

The following supporting information can be downloaded at: https://www.mdpi.com/article/10.3390/ijms24021681/s1.
