# Supplementary material for: A Single Nucleotide Polymorphism rs1010816 Predicts Sorafenib Therapeutic Outcomes in Advanced Hepatocellular Carcinoma
Source: Int J Mol Sci. 2023 Jan 14;24(2):1681. doi: 10.3390/ijms24021681 (PMC9862766; doi:10.3390/ijms24021681)
Supplement: Supplementary file 1 [file ijms-24-01681-s001.zip › ijms-2061680-supplementary.pdf]

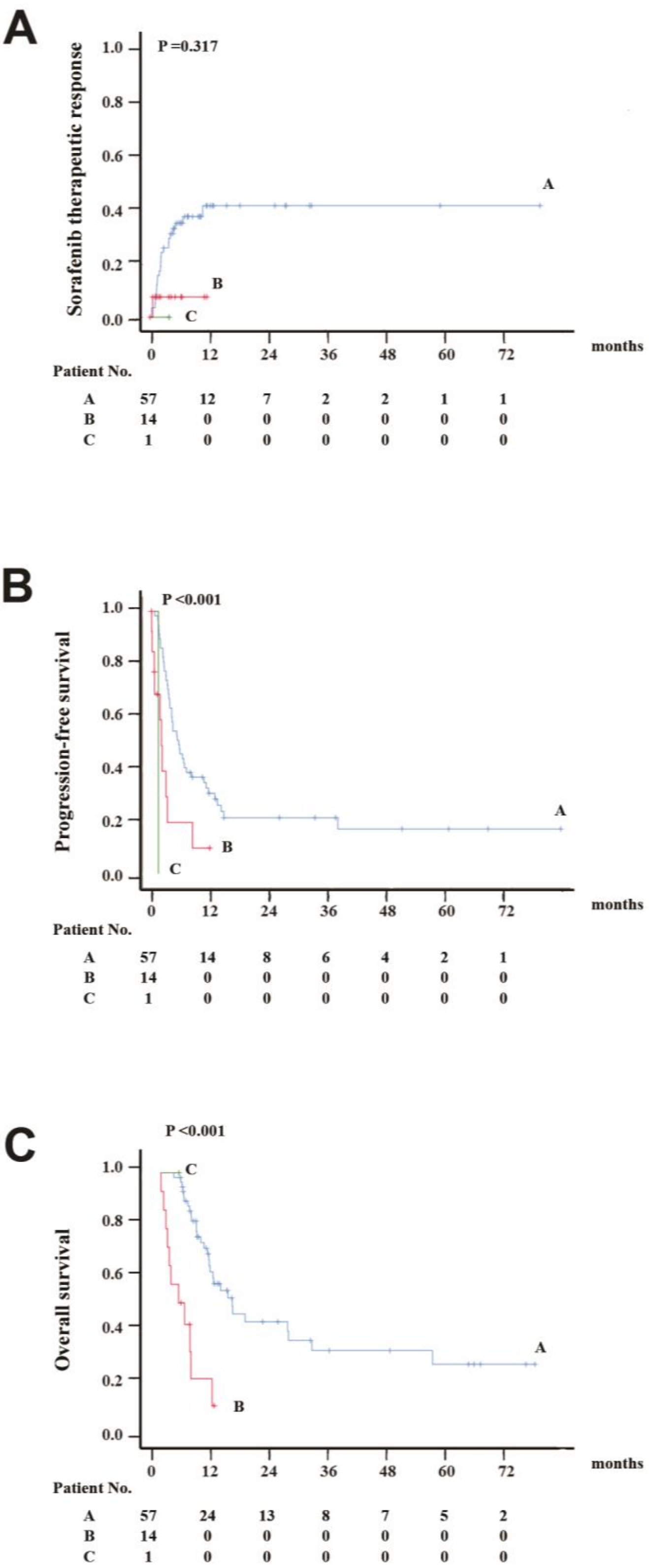

Supplementary Figure S1. Kaplan-Meier analysis in advanced HCC patients receiving sorafenib therapy. The clinical significances of Child-Pugh score on Sorafenib therapeutic response (**A**), progression-free survival (**B**) and overall survival (**C**) were analyzed by Kaplan-Meier method with log-ranked test.
